# Supplementary material for: Double dome structure of the Bose–Einstein condensation in diluted S = 3/2 quantum magnets
Source: Nat Commun. 2023 Mar 10;14:1260. doi: 10.1038/s41467-023-36725-4 (PMC10006222; doi:10.1038/s41467-023-36725-4)
Supplement: Supplementary file 1 — Supplementary Information [file 41467_2023_36725_MOESM1_ESM.pdf]

Supplementary Information

Double dome structure of the Bose–Einstein Condensation in Diluted $S = 3/2$ Quantum Magnets

Yoshito Watanabe,[1] Atsushi Miyake,[2] Masaki Gen,[1] Yuta Mizukami,[1] Kenichiro Hashimoto,[1] Takasada Shibauchi,[1] Akihiko Ikeda,[3] Masashi Tokunaga,[2,4] Takashi Kurumaji,[1] Yusuke Tokunaga,[1] and Taka-hisa Arima[1,4]

[1]*Department of Advanced Materials Science, The University of Tokyo, Kashiwa, 277-8561, Japan*

[2]*The Institute for Solid State Physics, The University of Tokyo, Kashiwa, 277-8581, Japan*

[3]*Department of Engineering Science, University of Electro-Communications, Chofu, 182-8585, Japan*

[4]*RIKEN Center for Emergent Matter Science (CEMS), Wako, 351-0198, Japan*

(Dated: February 9, 2023)

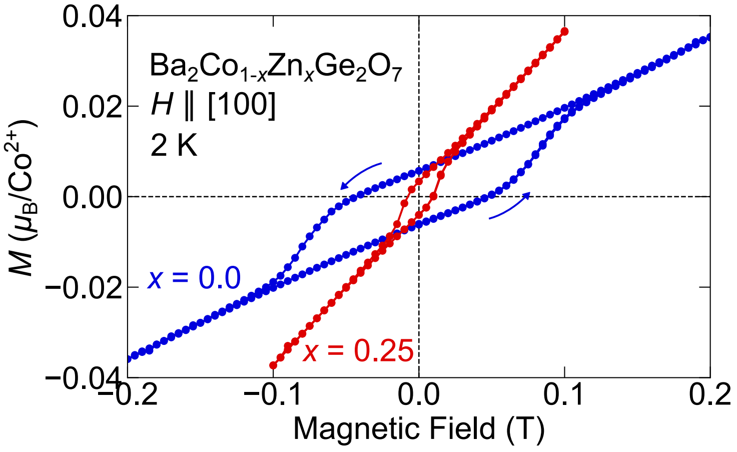

Suuplementary Fig. 1. **Weak ferromagnetic moment near the zero-field limit** for $Ba_2Co_{1-x}Zn_xGe_2O_7$.

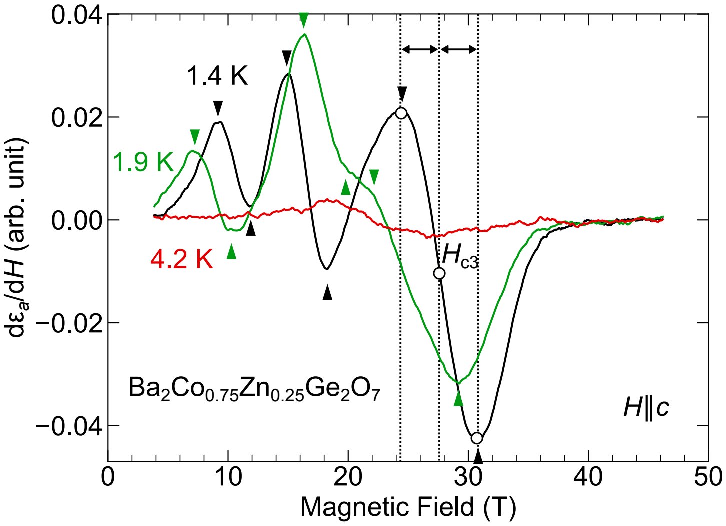

Suuplementary Fig. 2. **Field derivative of electric permittivity dε_a/dH for x = 0.25.** Peaks and dips in dε_a/dH–H curves for 1.4 (1.9) K are indicated by black (green) triangles.

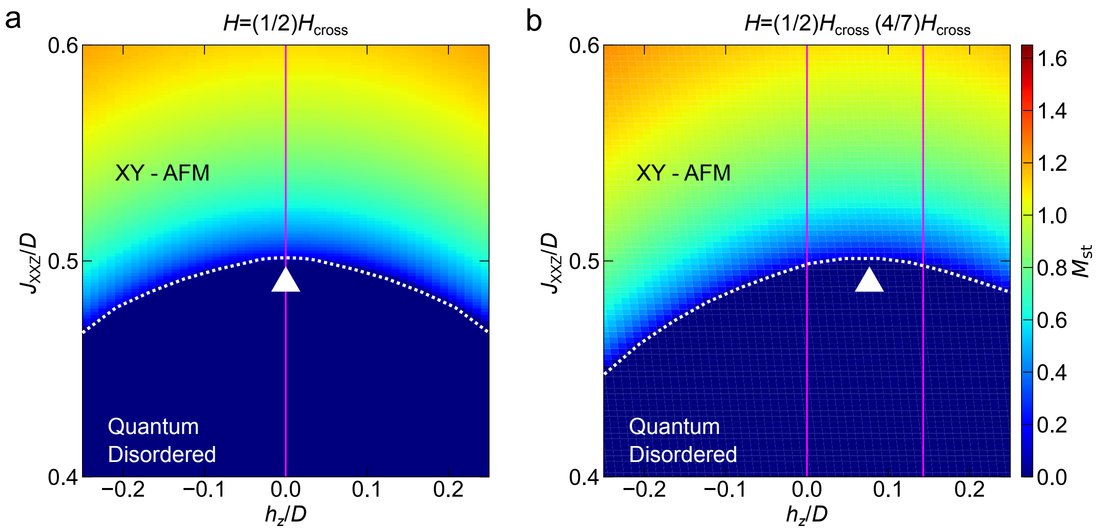

Suuplementary Fig. 3. **Effect of quadrupole terms.** (a) Magnetic phase diagram of the $S = 1$ effective spin model Eq. (3) drawn using the value of the staggered magnetization as an order parameter. (b) Magnetic phase diagram of the model which contains additional quadrupole $\hat{Q}_{zx}$ and $\hat{Q}_{yz}$ terms in Eqs. (3a) and (3b) in the main text. White dashed lines represent the phase boundary between quantum disordered phase and XY-antiferromagnetic phase.

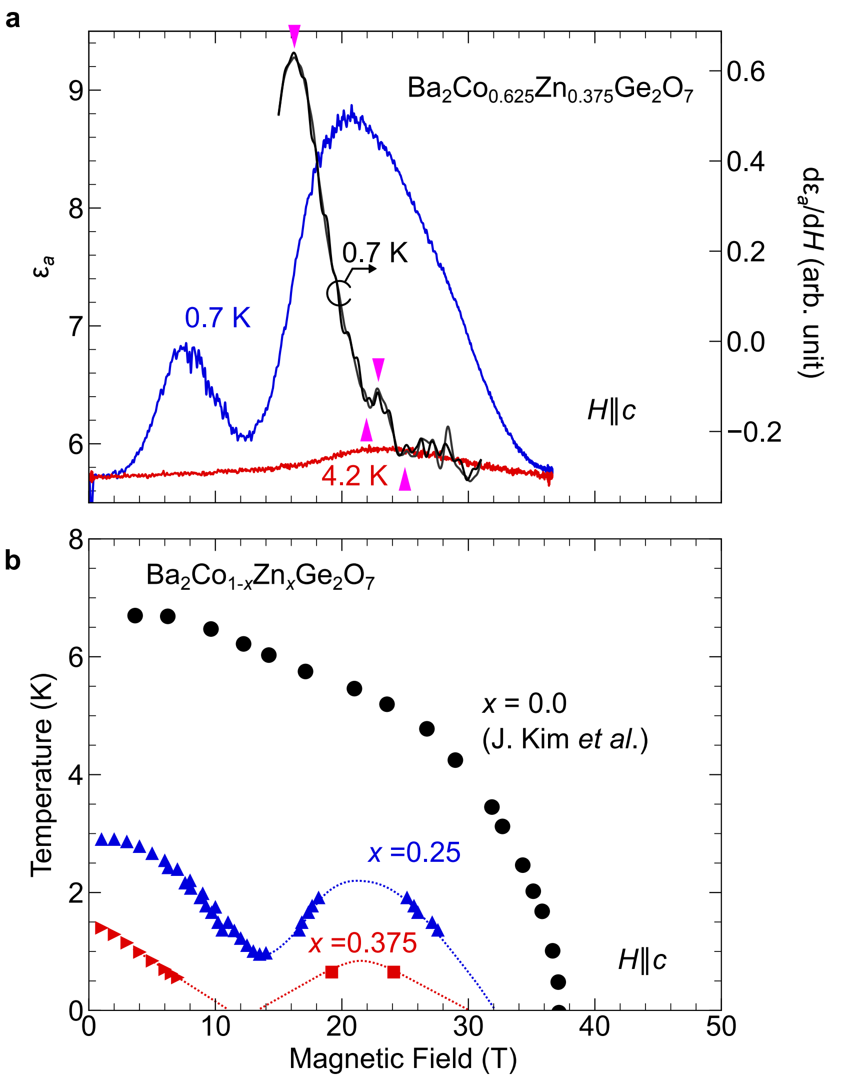

Suuplementary Fig. 4. **Extended magnetic phase diagram with possible phase boundaries for *x* = 0.375.** (a) Magnetic-field dependence of electric permittivity ε_a and its field derivative $\mathrm{d}\varepsilon_a/\mathrm{d}H$ for $x = 0.375$. The measurements at 0.7 K were performed twice, and we show both $\mathrm{d}\varepsilon_a/\mathrm{d}H$–$H$ curves, which are reproducible. The local minimum and maximum in the $\mathrm{d}\varepsilon_a/\mathrm{d}H$–$H$ curve used to obtain the critical fields H_{c2} and H_{c3} are shown by purple triangles. (b) Magnetic phase diagram for $x = 0$, 0.25 and 0.375. The black circles show the transition points of pristine $Ba_2CoGe_2O_7$, which are excerpts from Ref. [1].

Supplementary Note 1: Weak ferromagnetism in $Ba_2Co_{1-x}Zn_xGe_2O_7$

Due to the in-plane Dzyaloshinskii-Moriya interaction, the magnetic moments of Co ions are canted in the ab-plane from the collinear antiferromagnetic order [2]. Previous polarized neutron diffraction study suggests that the canting angle is less than 0.2° [3]. In Fig. 2b, we show the schematic of the magnetic structure and exaggerate the canting of the moment from the actual angle for visibility. In BCGO with magnetic ordering vector $\mathbf{k} = (1\ 0\ 0)$, zero-field spin canting causes a weak spontaneous magnetization in field dependence of magnetization [2]. As shown in Supplementary Fig. 1, we confirmed the residual magnetization at zero-field for BCZGO with $x = 0.25$ suggesting the small canting of Co ions moments. Therefore, we conclude the dilution process remains the zero-field magnetic structure intact. Note that, in the current study, among the possible directions of the weak ferromagnetic moment, the magnetic field would choose one configuration according to the finite misalignment of the magnetic field from the c-axis.

Supplementary Note 2: Estimation of critical field from magnetic field dependence of ε_a

To estimate critical fields from the experimental data of magnetic-field dependence of electric permittivity ε_a, we utilize the field derivative of electric permittivity dε_a/dH (Supplementary Fig. 2). At 1.4 K, there are three obvious peaks in the ε_c–H curve [Fig. 3 (e)], which are assigned to three critical fields H_{ci} ($i = 1, 2, 3$). Each peak in the ε_a–H curve is accompanied by a local maximum (minimum) on the lower (higher) field side in the dε_a/dH curve at 1.4 K, as indicated by black triangles. We regard the midpoint of the fields where the minimum or maximum appear as the critical field. The definition of H_{c3} at 1.4 K is depicted in Supplementary Fig. 2 for example. The critical fields determined in this way agree well with the lowest field peak in the temperature dependence of ε_a.

Supplementary Note 3: Effect of quadrupole terms to the phase diagram

If the effective models completely describe the evolution of the phase diagram of BCZGO, as we will soon see, the bottom of the valley between the two domes is precisely at the half of the field of the second dome's apex. As shown in Fig. 3, however, the value of $(1/2)H_{\text{cross}} \approx 11\,\text{T}$ roughly corresponds to H_{c1} at 1.4 K, which is in the middle of the downslope of AFM I rather than the bottom of the valley. This discrepancy could be explained by the effect of the quadrupole

terms in Eq. (3a) and (3b), which we omit when we derive the effective $S = 1$ Hamiltonian. Supplementary Figure 3 shows the results of the mean-field calculation of the $S = 1$ model with and without the quadrupole terms. We define the critical value $(J_{\rm XXZ}/D)_{\rm c}$ such that the system remains magnetically ordered for any values of h_z/D with a fixed value of $J_{\rm XXZ}/D$ larger than $(J_{\rm XXZ}/D)_{\rm c}$ but has an intermediate quantum disordered phase against h_z/D sweep for a smaller value of $J_{\rm XXZ}/D$ than $(J_{\rm XXZ}/D)_{\rm c}$. Regardless of the quadrupole terms, $(J_{\rm XXZ}/D)_{\rm c} \approx 0.5$, which is slightly smaller than 0.55 [4] obtained by quantum Monte Carlo simulation. At $(J_{\rm XXZ}/D)_{\rm c}$ and in the absence of the quadrupole terms, the magnetic order disappears at $h_z = 0$, marked by a white triangle in Supplementary Fig. 3 (a). Except for $h_z = 0$, or equivalently $H = (1/2)H_{\rm cross}$, the system shows ordering. In other words, the magnetic order becomes most unstable at $h_z = 0$, which makes a dip of the transition temperature at $h_z = 0$ in the magnetic phase diagram. As indicated by a white triangle in Supplementary Fig. 3 (b), quadrupole terms move the bottom of the valley toward positive h_z, i.e., $H > (1/2)H_{\rm cross}$. Since the first and second domes can be mapped on the effective $S = 1/2$ XXZ models centered at $H = 0$ and $H_{\rm cross}$, respectively, and its size is proportional to $J_{\rm XXZ}$, two domes merge near $H = (4/7)H_{\rm cross}$ but not at $H = (1/2)H_{\rm cross}$ when J increases. As J further increases, the dip structure in the phase diagram will be gradually smeared and moved to an even higher field; finally, the two domes are indistinguishable. Estimated $H = (4/7)H_{\rm cross}$ is also shown in Figs. 4 (a, b) and Supplementary Fig. 3.

Supplementary Note 4: Electric permittivity measurement in pulsed magnetic field for *x* = 0.375

Supplementary Figure 4 (a) shows the field dependence of ε_a for $x = 0.375$. The second peak in the ε_a–H curve suggests the existence of the second dome. Using the method used in $x = 0.25$, we add two points in the phase diagram for $x = 0.375$, as indicated by squares in Supplementary Fig 4 (b). However, unlike the ε_a–H curve at 1.4 K in $x = 0.25$, the H_c value estimated from $\mathrm{d}\varepsilon_a/\mathrm{d}H$ does not correspond to a clear peak but a shoulder in the ε_a–H curve. This is probably because the flatness of the top of the second dome makes it difficult to disentangle two domes even in the lowest available temperature. From the analogy with $x = 0.25$, we expect that the three-peak structure in ε_a–H becomes clearer for lower temperatures in $x = 0.375$. We note, however, that the nature of the second dome may deviate from the mean-field expectation, which relatively well explains the phase diagram of $x = 0.25$, owing to the stronger disorder effect in $x = 0.375$. An origin of such modification is the random-field nature of the effective $S = 1/2$

model in the high-field limit. As discussed in the main text, the position of the second dome centered around H_{cross} also depends on the J, which, in the current case, is randomly distributed over the system. Therefore, to elucidate the exact nature of the second dome in the high-doping limit such as the value of $H_{\mathrm{c}i}$ down to the absolute zero temperature, the temperature of the dome top, and the scaling exponent regarding the shape of the dome, we need further studies, such as an electric-permittivity measurement in a static magnetic field or for various x values, which is beyond the scope of the present study.

---

[1] J. W. Kim, S. Khim, S. H. Chun, Y. Jo, L. Balicas, H. T. Yi, S.-W. Cheong, N. Harrison, C. D. Batista, J. Hoon Han, and K. Hoon Kim, Manifestation of magnetic quantum fluctuations in the dielectric properties of a multiferroic, Nature Communications **5**, 4419 (2014).

[2] H. T. Yi, Y. J. Choi, S. Lee, and S. W. Cheong, Multiferroicity in the square-lattice antiferromagnet of $Ba_2CoGe_2O_7$, Applied Physics Letters **92**, 212904 (2008).

[3] V. Hutanu, A. P. Sazonov, M. Meven, G. Roth, A. Gukasov, H. Murakawa, Y. Tokura, D. Szaller, S. Bordács, I. Kézsmárki, V. K. Guduru, L. C. J. M. Peters, U. Zeitler, J. Romhányi, and B. Náfrádi, Evolution of two-dimensional antiferromagnetism with temperature and magnetic field in multiferroic $Ba_2CoGe_2O_7$, Physical Review B **89**, 064403 (2014).

[4] T. Roscilde and S. Haas, Mott Glass in Site-Diluted $S = 1$ Antiferromagnets with Single-Ion Anisotropy, Physical Review Letters **99**, 047205 (2007).
