# Supplementary material for: Double dome structure of the Bose–Einstein condensation in diluted S = 3/2 quantum magnets
Source: Nat Commun. 2023 Mar 10;14:1260. doi: 10.1038/s41467-023-36725-4 (PMC10006222; doi:10.1038/s41467-023-36725-4)
Supplement: Supplementary file 3 — Description of Additional Supplementary Files [file 41467_2023_36725_MOESM3_ESM.pdf]

File name: Supplementary Software 1

Description: Python script for finite temperature mean field calculations. The function `self_consistent_solver(field, temperature)` takes as input the magnetic field and the temperature, and returns the simulated thermal averages of the spin moment and the spin-induced electric dipole moment at sublattices *A* and *B*.
